# The Shifting Boundary Between Invasive and Non-Invasive Angiographic Investigation in Contemporary Cardiology and Cardiac Surgery: An Up-to-Date Narrative Review

**DOI:** 10.3390/jcm15145723

**Published:** 2026-07-21

**Authors:** Justin Ren, Colin Royse, William Chan, Dion Stub, Garry W. Hamilton, Jason E. Bloom, Tobias Fruehwald, Nilesh Srivastav, Alistair Royse

**Affiliations:** 1 Department of Surgery, The University of Melbourne, Melbourne, VIC 3010, Australia; 2 Department of Cardiothoracic Surgery, Royal Melbourne Hospital, Melbourne, VIC 3052, Australia; 3 Medical School, The University of Western Australia, Perth, WA 6009, Australia; 4 Department of Anaesthesia and Pain Management, Royal Melbourne Hospital, Melbourne, VIC 3052, Australia; 5 Outcomes Research, University of Texas Health, Houston, TX 77030, USA; 6 Department of Cardiology, Royal Melbourne Hospital, Melbourne, VIC 3052, Australia; 7 Baker Heart and Diabetes Institute, Melbourne, VIC 3004, Australia; 8 Department of Cardiology, Western Health, Melbourne, VIC 3011, Australia; 9 School of Public Health and Preventive Medicine, Monash University, Melbourne, VIC 3004, Australia; 10 Department of Cardiology, The Alfred Hospital, Melbourne, VIC 3004, Australia; 11 Department of Cardiology, Austin Health, Melbourne, VIC 3084, Australia; 12 Department of Medicine, University of Melbourne, Melbourne, VIC 3010, Australia; 13 Heart Vascular and Thoracic Institute, Cleveland Clinic Abu Dhabi, Abu Dhabi P.O. Box 112412, United Arab Emirates; 14 Department of Surgery, Universiti Kebangsaan Malaysia, Kuala Lumpur 56000, Malaysia

**Keywords:** coronary computed tomography angiography, CT-FFR, photon-counting CT, cardiac magnetic resonance, coronary artery bypass grafting, transcatheter aortic valve replacement, heart team, clinical decision-making

## Abstract

**Background:** Invasive coronary angiography has historically been the reference standard for coronary, valvular, and structural heart disease. Over the past decade, coronary computed tomography angiography (CCTA), CT-derived fractional flow reserve (CT-FFR), photon-counting detector computed tomography (PCCT), and cardiac magnetic resonance (CMR) have expanded the range of clinical questions answerable without an intra-arterial catheter, but this shift has been uneven across clinical domains. **Methods:** We performed a narrative review and synthesis of randomized trials, registries, society guidelines, and consensus documents (2009–2026) identified through PubMed and major cardiovascular guideline databases, written from a joint cardiology and cardiac-surgical standpoint. **Results:** The boundary has shifted asymmetrically, by which we mean a domain-dependent rather than uniform displacement of invasive angiography. Non-invasive imaging is now established as the first-line approach for stable chest pain at low-to-moderate pretest probability, for pre-transcatheter aortic valve replacement (TAVR) and structural procedural planning, and for aortic disease. It remains contested for stable multivessel disease and pre-coronary artery bypass grafting (CABG) planning, where CCTA- or CT-FFR-only planning is still investigational. Invasive angiography stays first-line for ST-elevation myocardial infarction (STEMI), cardiogenic shock, and complex percutaneous coronary intervention (PCI), where diagnosis and therapy are inseparable. **Conclusions:** Invasive and non-invasive modalities are complementary rather than competing. The appropriate first-line investigation depends on the disease domain, pretest probability, anatomical complexity, imaging quality, and whether diagnosis and treatment can be separated. We propose a complexity-stratified, heart-team framework and identify the surgical research gaps that remain.

## 1. Introduction

The diagnostic catheter and the therapeutic catheter were, until recently, the same instrument. F. Mason Sones introduced selective coronary cine-angiography in 1958, percutaneous coronary intervention (PCI) emerged in the late 1970s, and surgical revascularization developed in parallel [1,2]. The clinical environment that resulted answered two questions in the same room with the same wire: whether obstructive coronary disease was present, and what should be done about it. The coupling was efficient and durable, and it produced a working assumption that the catheter laboratory should be the first destination for almost any patient with suspected coronary, valvular, or aortic disease.

Over the past decade, this coupling has progressively decoupled. Multidetector and photon-counting CT, validated CT-derived fractional flow reserve (CT-FFR), stress cardiac magnetic resonance (CMR) perfusion, and AI-enabled plaque characterization have shifted a significant portion of the diagnostic workload upstream of the catheter laboratory. The therapeutic workload has remained within it. Diagnostic information about coronary circulation is now available along three partly independent axes: anatomy, lesion-level physiology, and plaque morphology. Each has both an invasive and a non-invasive counterpart, and the appropriate first investigation depends on which axis is most informative for the clinical question, not on a hierarchy among technologies. We use the term asymmetric shift to mean that this displacement of invasive angiography has advanced at very different rates across clinical domains.

The clinical yield of unselected diagnostic invasive coronary angiography (ICA) has long been modest. In the most-cited contemporary benchmark, only 37.6% of patients referred for elective diagnostic ICA were found to have obstructive coronary artery disease (CAD) [3]. More than a decade later, no large contemporary registry has shown that this yield has improved materially in centers that do not screen with non-invasive imaging. Reductions in unnecessary invasive testing therefore represent a legitimate quality goal rather than a downgrading of care, particularly in older patients with comorbidity in whom the cumulative burden of contrast nephropathy, access-site complications, and procedural delay is often significant.

The evidence driving this redistribution has, however, been generated predominantly by interventional and imaging cardiology investigators. Few of the major randomized trials shaping current practice have had cardiac-surgical principal investigators, surgical primary endpoints (graft patency, completeness of revascularization, conduit selection, intraoperative plan modification), or surgical candidates as the index population. Cardiac surgery, which will be most affected operationally by any change in pre-procedural workflow, has therefore had limited contribution to the evidence behind that change. This narrative review is written from a joint cardiology and cardiac-surgical standpoint. We describe where current evidence supports a non-invasive first investigation, where ICA remains the appropriate starting point, where the evidence is still being assembled, and where the gap between trial cohorts and real-world clinical practice will determine whether the framework can actually be delivered.

## 2. Methods

This article is a narrative review and synthesis intended to integrate contemporary evidence and to propose a practical, heart-team decision framework rather than to provide a systematic appraisal. We searched PubMed/MEDLINE and the Cochrane Library for English-language randomized controlled trials, large registries, meta-analyses, current society guidelines, and expert-consensus documents. Search terms combined the principal diagnostic modalities, including coronary CT angiography, CT-derived fractional flow reserve, photon-counting CT, cardiac magnetic resonance, ICA, fractional flow reserve, and intracoronary imaging, with the main clinical domains, including transcatheter aortic valve replacement, coronary artery bypass grafting, and heart-team decision-making. We concentrated on literature from the contemporary multidetector CT era, broadly the past fifteen years, while retaining earlier landmark studies that remain clinically foundational, and we hand-searched the reference lists of key articles for additional sources. Studies were selected by author consensus on the basis of clinical relevance and methodological quality, with randomized outcome data given the greatest weight, followed by registry and feasibility data and then expert opinion. The strength of the evidence underpinning each statement is indicated in the relevant section.

## 3. Coronary Artery Disease

### 3.1. Stable Chest Pain Pathways

In chest pain at low-to-moderate pretest probability, the evidence supporting CCTA as the appropriate first-line investigation is now extensive and consistent across geographies. Newby and colleagues (Table 1), in the Scottish SCOT-HEART trial (n = 4146), reported a 41% reduction in the composite of coronary heart disease death or non-fatal myocardial infarction (MI) at five years (2.3% vs. 3.9%; hazard ratio [HR] 0.59, 95% confidence interval [CI] 0.41 to 0.84) without an increase in invasive procedures, the only CCTA-led care trial to date to show a hard-event signal of this magnitude [4,5]. The DISCHARGE trial (n = 3561) subsequently established non-inferior major adverse cardiovascular events (MACE) at 3.5 years against an ICA-first strategy in patients with intermediate pretest probability, with major procedure-related complications halved from 1.9% to 0.5% [6]. In PROMISE (Douglas and colleagues, n = 10,003), CCTA produced clinical outcomes equivalent to functional testing while reducing downstream catheterizations that found no obstructive disease [7]. CONSERVE reported an approximately 78% reduction in ICA without obstructive CAD when CCTA gated the referral pathway [8]. PRECISE combined the PROMISE Minimal Risk Score (PMRS) with CCTA and reduced its composite primary endpoint by 71% (adjusted HR, 0.29; 95% CI, 0.20–0.41; *p*  <  0.001); this reduction was driven almost entirely by the low-yield catheterization component (10.2% to 2.6%), with no difference in death or myocardial infarction [9]. PRECISE therefore supports risk-stratified CCTA as a means of reducing low-yield invasive testing rather than as a mortality intervention.

Cardiac magnetic resonance (CMR) perfusion offers a validated non-invasive functional alternative. In MR-INFORM, Nagel and colleagues reported non-inferior MACE at one year against an invasive fractional flow reserve (FFR)-guided strategy, with fewer revascularizations performed in the CMR arm [10]. CMR is less sensitive than FFR at the lesion level, but the lesions it does not flag did not translate into a measurable event difference at this time horizon. The 2021 AHA/ACC chest pain guideline gives CCTA a Class I (Level A) recommendation for intermediate-to-high-risk patients with stable chest pain and no known CAD, and the 2024 ESC chronic coronary syndromes guideline recommends CCTA (Class I) as a first-line test in patients with low-to-moderate clinical likelihood of obstructive CAD [11,12].

The translation of this evidence into routine practice has been uneven, and most clinicians will recognize a gap between trial recommendations and what is realistically available in a typical outpatient cardiology service. CCTA throughput remains dependent on local scanner specifications, reader expertise, and dedicated reporting time, all of which vary substantially between tertiary centers and community programs. CT-FFR requires off-site processing with a turnaround that, in most health systems, exceeds 24 h, and reimbursement is not yet uniform. In settings where CCTA cannot be delivered within a clinically reasonable interval or where reads are inconsistent, the practical default still drifts toward direct ICA despite the evidence.

### 3.2. Planning Revascularization

**Figure 1:**
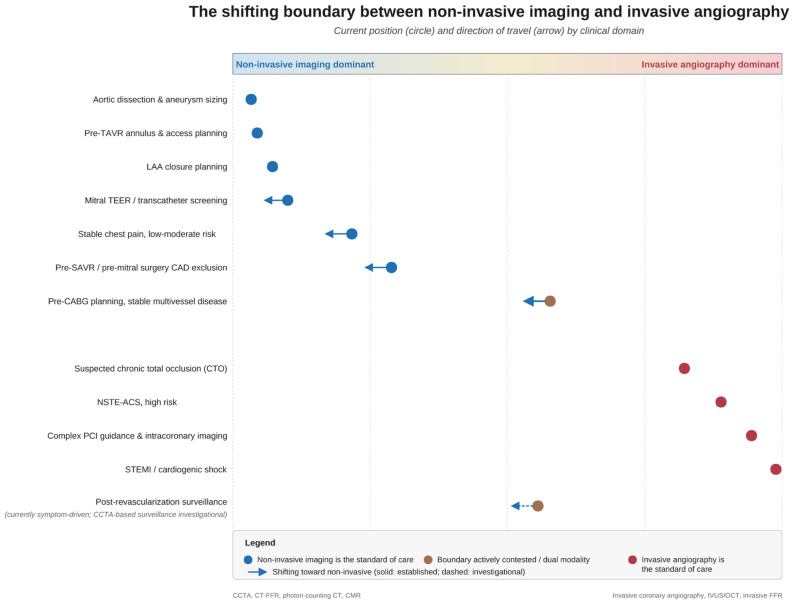
Position of non-invasive imaging and invasive angiography across clinical domains. Circles mark current position on the spectrum; arrows mark direction of recent evidence-based movement. The boundary has moved toward non-invasive imaging in aortic disease, pre-TAVR planning, left atrial appendage closure planning, and pre-mitral structural intervention. It is contested in stable multivessel coronary disease and pre-CABG planning. It remains predominantly invasive in STEMI, cardiogenic shock, and complex PCI guided by intracoronary imaging. CCTA, coronary computed tomography angiography; CT-FFR, CT-derived fractional flow reserve; PCCT, photon-counting CT; CMR, cardiac magnetic resonance; LAA, left atrial appendage; TEER, transcatheter edge-to-edge repair; SAVR, surgical aortic valve replacement; TAVR, transcatheter aortic valve replacement; CABG, coronary artery bypass grafting; CTO, chronic total occlusion; NSTE-ACS, non-ST-elevation acute coronary syndrome; STEMI, ST-elevation myocardial infarction; ICA, invasive coronary angiography.

The role of non-invasive imaging in revascularization planning is the most actively contested part of the boundary: CCTA-first evaluation of stable chest pain and CT-based TAVR planning are established, guideline-endorsed standards, whereas CCTA/CT-FFR-only pre-CABG planning remains investigational and should currently be confined to research protocols (Figure 1). The ISCHEMIA trial (Maron, Hochman, and colleagues, n = 5179) used blinded CCTA as an anatomic screen for randomization in approximately three-quarters of enrolled patients, principally to exclude unprotected left main disease and to confirm obstructive coronary anatomy. The primary comparison, however, was strategy-level (invasive versus conservative initial management) rather than between imaging modalities. ISCHEMIA’s anatomic gatekeeping by CCTA was structurally important because it signaled that trialists accepted CCTA as adequate to confirm enrollment-grade disease. It was not, however, a randomized test of CCTA against ICA for revascularization planning [13].

Two more recent studies address preoperative imaging in surgical candidates more directly. In BYPASS-CTCA, Jones and colleagues randomized 688 patients with prior coronary artery bypass grafting (CABG) referred for ICA to CCTA before invasive angiography or standard care, reporting shorter procedure time, lower contrast volume, and a reduction in additional invasive testing in the imaging-first arm; CCTA can streamline a planned invasive procedure but does not replace it [14]. In FASTTRACK CABG (Serruys and colleagues, n = 114), surgical revascularization was planned entirely from CCTA and CT-FFR in stable multivessel disease without preoperative ICA. Surgeons judged the imaging-only plan executable in 99% of cases, with 30-day outcomes consistent with contemporary CABG benchmarks [15]. The trial is a feasibility signal, not a definitive comparison. FASTTRACK CABG was a single-arm study of 114 highly selected, low-surgical-risk patients, with a surgeon-adjudicated feasibility endpoint and follow-up limited to 30-day CCTA graft assessment. Its findings may not extend to patients underrepresented in or excluded from the trial—those with diffuse disease, severe calcification (where blooming artifact degrades assessment of distal targets), prior PCI, chronic kidney disease, or diabetes. A properly powered randomized comparison with surgical primary endpoints (graft patency, completeness of revascularization, intraoperative plan modification) is required before CCTA-only planning can be recommended as a routine alternative to ICA in surgical candidates.

ISCHEMIA-EXTEND, the extended observational follow-up at a median of 5.7 years, adds nuance. It reported lower seven-year cumulative cardiovascular mortality with the invasive strategy (6.4% vs. 8.6%; adjusted HR 0.78), although all-cause mortality did not differ and non-cardiovascular mortality was higher with invasive management [16]. These are non-prespecified exploratory analyses and should be read as hypothesis-generating. Their relevance is that the imaging modality is only one element of a broader decision about whether and when to revascularize.

In contemporary multidisciplinary practice, the decision to plan CABG without preoperative ICA is rarely a fixed threshold, but rather, a judgment shaped by available imaging quality, local surgical comfort, and patient-specific risk. For a stable 65-year-old with low-to-moderate-complexity disease and a high-quality CCTA in a well-resourced center, an imaging-only pathway is increasingly defensible. For a 78-year-old with prior PCI, multivessel disease, and partial calcification, the threshold for ICA remains low even in centers comfortable with imaging-first triage. Imaging-only planning therefore depends not only on the evidence base but on the consistency of imaging quality that the surgical team can actually rely on.

### 3.3. Invasive Physiology and Intracoronary Imaging

Invasive coronary investigation has not narrowed but has been refined. FAME-3 randomized 1500 patients with three-vessel disease to FFR-guided PCI or CABG; at one year, the primary composite of death, MI, stroke, or repeat revascularization occurred in 10.6% of the PCI arm and 6.9% of the CABG arm (HR 1.5, 95% CI 1.1 to 2.2). FFR-guided PCI did not meet the prespecified non-inferiority margin against CABG, with results favoring CABG and supporting the continued role of surgical revascularization in three-vessel disease [17]. Intracoronary imaging-guided PCI is now differentiated by context. ILUMIEN-IV (n = 2487) compared optical coherence tomography (OCT)-guided with angiography-guided PCI in a broad population and reported larger minimum stent area with OCT guidance and lower stent thrombosis, but no significant reduction in target-vessel failure at two years [18]. In contrast, RENOVATE-COMPLEX-PCI, restricted to complex lesions and comparing imaging-guided (OCT or intravascular ultrasound [IVUS]) with angiography-guided PCI, reduced a composite of cardiac death, target-vessel MI, and clinically driven target-vessel revascularization (7.7% vs. 12.3% at median 2.1 years; HR 0.64) [19]. The clinical benefit of imaging guidance is confined to complex disease and is not apparent in unselected PCI populations. The OPTIMAL trial similarly found that intravascular ultrasound-guided PCI of unprotected left main disease did not improve outcomes over angiography-guided treatment at a median of 2.9 years [20]. iFR-SWEDEHEART and DEFINE-FLAIR established the instantaneous wave-free ratio (iFR) as non-inferior to FFR for MACE at one year and removed the adenosine barrier [21,22]. Read together, invasive physiology provides clinical benefit in stable disease and elective lesion selection but does not consistently outperform angiographic guidance in every setting.

### 3.4. Advances in Technology and Their Current Limits

CT-FFR has been validated against invasive FFR. Nørgaard and colleagues, in the NXT analysis, reported per-patient sensitivity of 86%, specificity of 79%, and area under the receiver-operating-characteristic curve of 0.90 in patients with interpretable images [23]. Downstream signals from PLATFORM and the ADVANCE registry are also positive: the CT-FFR pathway reduced the proportion of patients referred to ICA without obstructive disease from 73% to 12% in PLATFORM, and clinical management changed in approximately 67% of patients after CT-FFR became available across the ADVANCE cohort [24,25]. Curzen and colleagues, in the pragmatic open-label UK FORECAST trial of 1400 patients with stable chest pain, showed no significant reduction in cardiac costs or clinical events at nine months, despite a modest reduction in invasive angiography [26], suggesting that the technology may not perform identically in routine (“real world”) practice as it does in curated referral cohorts. The divergence from PLATFORM and ADVANCE reflects design differences: FORECAST was open-label and pragmatic, with CT-FFR placed earlier in the diagnostic pathway and a less curated patient population. Real-world performance is also sensitive to image quality, calcium burden, motion artifact, and segmentation accuracy, and agreement with invasive FFR falls in heavily calcified disease; CT-FFR is therefore best regarded as an adjunct to high-quality CCTA rather than a universal replacement for invasive physiological assessment.

Photon-counting CT (PCCT) offers higher spatial resolution and reduced blooming. Early head-to-head work by Si-Mohamed, Mergen, and colleagues shows diagnostic accuracy approaching that of ICA in patients with moderate-to-high calcium burden [27,28], though it is not yet in widespread clinical use.

CCTA-derived plaque characterization provides prognostic information that lumen-based imaging alone cannot. Oikonomou and colleagues showed in CRISP-CT that the pericoronary fat attenuation index (FAI) predicts cardiac mortality independent of stenosis severity, and large registry analyses are extending these findings to plaque progression [29]. Total plaque burden and low-attenuation plaque volume contribute additional risk-stratification information [30,31,32]. Comparable wall-based information is obtainable invasively by OCT or IVUS at the lesion of interest, but the two approaches are complementary: CCTA provides whole-tree non-invasive assessment, while intracoronary imaging provides higher-resolution lesion-level detail at the point of decision. Plaque-based prognostication is the area in which non-invasive imaging offers information that invasive measurement structurally cannot deliver across the whole coronary tree.

### 3.5. Acute and Procedural Settings

**Figure 2:**
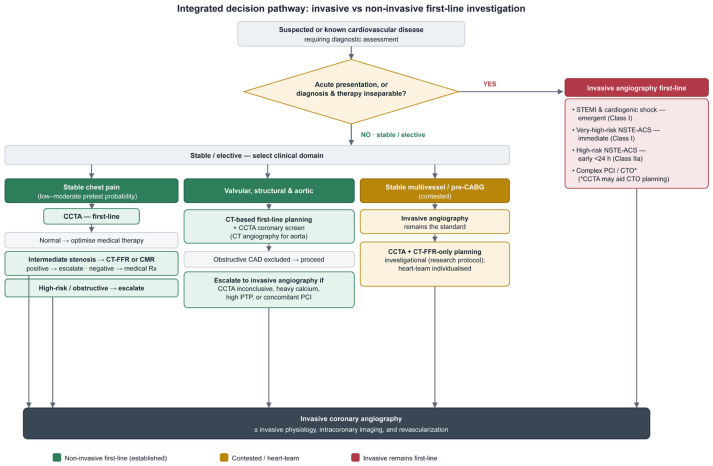
Integrated decision pathway for first-line invasive versus non-invasive investigation in suspected or known cardiovascular disease. Patients are triaged first by whether the presentation is acute or diagnosis and therapy are inseparable, favoring invasive angiography as first-line and by clinical domain otherwise. Recommendation classes follow the ESC acute coronary syndrome and ESC chronic coronary syndrome guidelines. The pathway reflects a deliberately CT-first framing: stress/functional imaging is an accepted alternative first-line option at intermediate-to-high clinical likelihood, and deferral of testing may be appropriate at genuinely low likelihood. CABG, coronary artery bypass grafting; CAD, coronary artery disease; CCTA, coronary CT angiography; CMR, cardiovascular magnetic resonance; CT-FFR, CT-derived fractional flow reserve; CTO, chronic total occlusion; NSTE-ACS, non-ST-elevation acute coronary syndrome; PCI, percutaneous coronary intervention; STEMI, ST-elevation myocardial infarction.

For acute coronary syndromes and complex coronary intervention, ICA remains the appropriate initial investigation (Figure 2) because the diagnostic and therapeutic decisions are inseparable from the procedure. The 2023 ESC acute coronary syndromes guideline [33] recommends immediate invasive angiography in very-high-risk non-ST-elevation acute coronary syndromes (NSTE-ACS), an early invasive strategy within 24 h in high-risk NSTE-ACS (Class IIa), and emergent ICA in STEMI and cardiogenic shock [34]. The main place non-invasive imaging contributes here is procedural planning; for example, chronic total occlusion (CTO) management benefits from CCTA, including lesion length, calcification, and collateral architecture, but the procedural decision and the intervention itself remain invasive [35]. Even within the invasive procedure, physiology is refining lesion selection that Xu and colleagues, in the sham-controlled FAVOR III China trial [36], showed that a quantitative flow ratio (QFR)-guided strategy reduced one-year MACE compared with angiography-guided lesion selection. In patients in whom revascularization itself confers no benefit over optimal medical therapy, as shown by Bangalore and colleagues in ISCHEMIA-CKD and by Perera and colleagues in REVIVED-BCIS2 (advanced kidney disease and ischemic left ventricular [LV] dysfunction, respectively) [37,38], the choice between invasive and non-invasive imaging becomes largely irrelevant, because it is downstream of the more fundamental decision of whether to revascularize at all, and is rarely on the critical path.

### 3.6. Coronary Angiography Without Obstructive Coronary Disease

In a subset of patients with anginal symptoms and unobstructed coronary arteries, ischemia is mediated by microvascular dysfunction or vasospasm rather than by epicardial stenosis. Ford and colleagues, in CorMicA (n = 151), randomized such patients to a stratified diagnostic protocol (acetylcholine provocation, coronary flow reserve, and microvascular resistance measurement performed at the time of angiography) or a sham-disclosure control. The intervention arm achieved an 11.7-point greater improvement on the Seattle Angina Questionnaire summary score at six months, exceeding the commonly cited minimal clinically important difference [39]. CorMicA is predominantly a single-center Scottish trial of modest size with a six-month endpoint and requires replication in larger and more diverse cohorts. The catheter laboratory’s scope is also widening. Where the angiogram alone would once have been read as negative, invasive functional and provocative testing can now establish a diagnosis. In this sense, the invasive lab has become the downstream counterpart of the upstream shift of anatomic diagnosis into non-invasive imaging.

## 4. Valve Disease

In valvular heart disease, non-invasive imaging has largely supplanted diagnostic catheterization, but two distinct roles of CT before transcatheter aortic valve replacement (TAVR) must be separated: procedural planning, which is an established standard, and CAD assessment in place of routine ICA, which is emerging but not yet supported by randomized strategy-level evidence. Multidetector CT before TAVR, which assesses annular dimensions, coronary ostial heights, iliofemoral access, and calcium distribution, has been the guideline-endorsed standard of care for more than a decade [40,41]. CT planning now anticipates complications as well as sizing: sinus and sino-tubular-junction dimensions and the valve-to-coronary distance identify patients at risk of coronary obstruction, a risk that is highest in valve-in-valve procedures and can be mitigated by intentional leaflet laceration (BASILICA) or commissural alignment to preserve coronary access [42,43,44].

Whether the same acquisition can also replace routine ICA for CAD assessment is a separate question, and the evidence here is accuracy- and cohort-level rather than strategy-level. A meta-analysis of 27 studies [45] (7458 TAVI candidates) reported patient-level sensitivity of 95% and negative predictive value of 97% for CCTA against ICA, but specificity of 73% and positive predictive value of 64%. This is a profile suited to ruling out obstructive disease rather than confirming it, and one that degrades with the heavy calcification typical of elderly aortic stenosis patients. Observational selective-ICA pathways support feasibility: in a cohort of 240 TAVI candidates triaged by CCTA, ICA was canceled in 80%, with no difference in major adverse cardiovascular events at a median of 15 months [46]. No randomized trial, however, has compared a CCTA-based selective-ICA pathway against a routine ICA-containing work-up on clinical outcomes. Guidance has nonetheless moved ahead of randomized evidence with the 2025 ESC/EACTS guidelines now recommending CCTA to rule out CAD in valve patients with low-to-moderate (≤50%) pretest likelihood of obstructive disease and stating that omission of ICA should be considered when the standard pre-TAVI CT is of sufficient quality to exclude relevant CAD [47].

The same logic extends to surgical candidates. CCTA is an accepted first-line coronary assessment before SAVR and mitral surgery at low-to-moderate pretest probability, supported by meta-analytic data and the 2025 ESC/EACTS and 2020 ACC/AHA guidelines [47,48,49]. Across both transcatheter and surgical populations, invasive angiography remains preferred when pretest probability is high, calcification or image quality precludes confident coronary evaluation, ischemic mitral regurgitation is suspected, intermediate stenoses require physiological assessment that CT-FFR cannot resolve, or revascularization is planned.

The management of obstructive CAD detected before TAVR is no longer entirely unresolved. In the randomized NOTION-3 trial, FFR-guided PCI in TAVI patients with significant CAD reduced the composite of all-cause death, myocardial infarction, or urgent revascularization compared with conservative management (median follow-up 2 years), at the cost of more minor bleeding [50]. Questions of timing, completeness of revascularization, and generalizability to higher-risk anatomy remain open.

Delivering a CCTA-first pathway in this population is now chiefly an implementation problem, requiring dedicated cardiac CT reading expertise integrated with the structural heart team rather than reading delegated to general radiology.

## 5. Structural Heart Disease

For transcatheter structural intervention, multidetector CT has effectively replaced angiography for procedural planning. Pre-WATCHMAN and pre-Amplatzer Amulet CT for left atrial appendage (LAA) closure are now standard in high-volume programs and are used alongside or in place of transesophageal echocardiography (TEE) for ostial sizing [51]. Patient-specific computational simulation is increasingly used to model device-anatomy interaction and to guide sizing for left atrial appendage closure and valve-in-valve procedures [52]. Mitral transcatheter edge-to-edge repair (TEER) and transcatheter mitral valve replacement screening use three-dimensional CT to assess leaflet anatomy, calcium distribution, and neo-left ventricular outflow tract (neo-LVOT) geometry, where a small predicted neo-LVOT identifies patients at high risk of iatrogenic outflow-tract obstruction, particularly in the setting of mitral annular calcification [53,54,55]. Transcatheter tricuspid repair and replacement rely similarly on CT and echocardiographic characterization of annular dimensions, leaflet morphology, and right coronary artery proximity [56]. Invasive hemodynamic catheterization of the right and left heart, however, remains important for risk stratification in mixed valve disease and heart failure, where filling pressures, pulmonary vascular resistance, and transvalvular gradients provide prognostic information that CT does not replicate. Concomitant coronary assessment in transcatheter mitral pathways continues to require ICA when CCTA cannot exclude obstructive disease with adequate confidence. CT has replaced angiography for procedural planning in this domain, while invasive hemodynamic catheterization remains complementary rather than displaced.

## 6. Aortic Disease

For aortic disease, the boundary has moved completely. CT angiography is the first-line imaging modality for the diagnosis and surveillance of aortic dissection, intramural hematoma, and thoracic and abdominal aortic aneurysm; CMR provides an alternative without ionizing radiation for surveillance of younger patients with connective tissue disease [57,58]. The role of ICA in aortic disease is now largely procedural, encompassing fluoroscopic deployment of endovascular grafts and concomitant coronary assessment before open or hybrid aortic repair. The 2022 ACC/AHA aortic disease guideline reflects this distribution [59].

## 7. Settings in Which ICA Remains First-Line

Several scenarios are not candidates for non-invasive substitution of ICA. In each, the diagnostic and therapeutic decisions are inseparable from the procedure itself: STEMI and cardiogenic shock (immediate invasive assessment, any pre-procedural imaging delays treatment); high-risk NSTE-ACS (invasive evaluation within 24 h per guideline); suspected CTO (CCTA aids planning but the procedural decision is invasive); and complex multivessel or high-anatomical-complexity PCI guided by intracoronary imaging and invasive physiology. A second category fails CCTA on diagnostic-confidence grounds rather than acuity: heavy coronary calcification, persistent tachyarrhythmia, or body habitus that prevents diagnostic image quality [26]. The relevance of this category will shift as PCCT matures, but in current practice it remains a defined indication for ICA. Across these scenarios, the shift toward non-invasive imaging in diagnostic triage does not displace the role of ICA in procedural or time-critical contexts; the two roles are distinct and not in competition.

## 8. The Operative Envelope: What CCTA Does Not Yet Deliver to the Surgeon

CCTA does not currently provide several pieces of operative information that cardiac surgeons routinely act on, and underemphasizing this distorts the imaging-versus-invasive debate as it applies to CABG candidates. The four observations below are drawn from operative experience and specify the envelope within which any imaging-only preoperative strategy would need to be validated. These observations reflect expert surgical opinion and graft-patency data rather than randomized evidence.

First, the in vivo pliability and intramural calcification distribution of the distal target vessel are assessed at operation by direct palpation and inspection. For vessels with a caliber below approximately 1.5 mm, this information is difficult to infer from any current imaging modality. This threshold, derived from the authors’ operative experience and consistent with published graft-patency literature [60,61], marks the point at which both CCTA and ICA show degraded predictive value for graft suitability. Second, dynamic distal run-off under pulsatile load, which informs the choice of conduit among the left internal mammary artery, the radial artery, and the saphenous vein, is a physiological judgment that is not currently captured by CT-FFR. Third, epiaortic and porcelain-aorta calcification, while increasingly resolvable on photon-counting CT, is best confirmed intraoperatively by epiaortic ultrasound, which has higher sensitivity than transesophageal echocardiography or preoperative CT for clinically significant ascending aortic atheroma and directly influences cross-clamp positioning, cannulation strategy, and the choice of on-pump or off-pump approach [62,63]. Fourth, the granular information required for second-order and third-order grafting decisions in diffuse multivessel disease is acquired intraoperatively, when the surgeon weighs target quality, run-off, conduit length, and intrathoracic anatomy together.

FASTTRACK CABG illustrates the practical edge of this envelope. The imaging-only plan was judged executable in 99% of cases, yet surgeons modified the planned distal target, conduit, or graft sequence intraoperatively in approximately one in six (16%) patients after direct inspection [15]. These findings should be interpreted with caution given the trial’s single-arm design and highly selected, low-surgical-risk cohort; the 16% modification rate may rise in less selected populations, an empirical question that should be tested in a trial. Because each of the surgical limitations set out above implies a specific, measurable endpoint, the operative envelope can be operationalized rather than merely asserted. A confirmatory trial should enroll within a defined complexity range (for example, SYNTAX score ≤ 22 [64,65], well-defined distal targets, absence of diffuse heavy calcification) and prespecify a surgically meaningful endpoint set, including target-vessel suitability, conduit-selection modification, completeness of revascularization, change in cannulation or cross-clamp strategy, graft patency at defined follow-up, and the need for rescue (bail-out) invasive angiography, with total intraoperative plan modification as the primary or co-primary endpoint. Defining these endpoints a priori keeps trial designs from ignoring the operative envelope and generating data that surgeons cannot apply.

## 9. Discussion

The strength of the evidence varies markedly by domain. CCTA-first evaluation of stable chest pain (supported by randomized outcome trials such as SCOT-HEART, PROMISE, and DISCHARGE) and CT-based procedural planning for TAVR (a guideline-endorsed standard of care) rest on a firmer evidence foundation than CCTA/CT-FFR-only CABG planning or routine photon-counting CT in heavily calcified disease, which remain supported mainly by feasibility and early comparative data. CCTA-based coronary assessment to replace routine invasive angiography before TAVR occupies an intermediate position: now guideline-endorsed at low-to-moderate (≤50%) pretest likelihood but not yet tested against routine invasive angiography in a randomized strategy-level trial. We therefore group clinical scenarios into three categories—non-invasive first-line, contested or heart-team individualized, and invasive first-line—that map directly onto the graphical abstract and Table 2.

Non-invasive imaging offers lower procedural risk, the ability to characterize plaque and extracardiac structures, and efficient triage away from low-yield catheterization, whereas invasive angiography offers definitive spatial resolution, on-table physiology, intracoronary imaging, and the option of immediate treatment. The limitations of non-invasive imaging extend beyond the surgical considerations discussed above: arrhythmia or high and irregular heart rates, renal dysfunction and contrast exposure, obesity and blooming artifact from dense calcification, incidental extracardiac findings, over-calling of intermediate stenoses and the downstream testing it generates, and variable reader expertise all constrain real-world performance.

These considerations make implementation, not evidence alone, decisive. An imaging-first pathway depends on scanner availability and generation, CT-FFR processing turnaround, reimbursement, dedicated reader expertise, and integration with the heart team, capabilities that remain concentrated in tertiary centers. Distinguishing what is technically possible from what is routinely deliverable across different health systems is therefore central to applying any framework and is the lens through which the future directions below should be read.

## 10. Future Directions

The direction of advancement is reasonably clear, even if its pace is not. As multidetector CT, CT-FFR, and photon-counting CT continue to mature, the diagnostic workload is likely to move further upstream of the catheter laboratory, and ICA is likely to become a less frequent first-line diagnostic test in selected stable, low-complexity domains, while remaining diagnostically essential in many real-world settings, including heavy calcification, prior revascularization, limited image quality, complex anatomy, acute presentation, invasive physiology, and constrained access. Several questions must be resolved before that shift can be endorsed for the surgical patient.

The most clinically consequential is whether CCTA and CT-FFR-only preoperative planning can become a routine alternative to ICA-based planning in stable multivessel coronary disease. FASTTRACK CABG provided a 114-patient feasibility signal [15], but a properly powered randomized comparison is required. The diagnostic performance of CCTA in heavily calcified disease and in the assessment of patent grafts overlying native vessels remains thin; head-to-head comparisons of PCCT with ICA in patients with high Agatston scores or prior CABG would clarify its role [27,28,66]^.^ Post-revascularization surveillance is currently driven by symptoms and stress testing, and whether longitudinal CCTA with quantitative plaque analysis identifies graft vulnerability or stent-edge progression early enough to change outcomes has not been tested in a randomized comparison [31,32].

Three contextual factors cut across these gaps. Access to CCTA, CT-FFR, and AI plaque-quantification infrastructure is heterogeneous globally, and an imaging-first approach risks replicating existing disparities in cardiovascular care [67]. Cost-effectiveness data are reassuring for CCTA in stable chest pain pathways but more limited for the pre-CABG and pre-valve indications [8,68]. The trial populations underlying much of this evidence appear predominantly European and North American, with limited reported representation of Black, Hispanic, South Asian, and East Asian patients, and women have generally been underrepresented in invasive physiology and intracoronary imaging trials. These observations reflect the available literature rather than a formal analysis, and the magnitude of these disparities and their effect on outcomes warrant dedicated study. Standardized structured reporting, including CAD-RADS 2.0, is essential for consistent implementation at scale [69], and future randomized comparisons would benefit from enrolling more geographically and demographically diverse cohorts as a design priority.

## 11. Conclusions

The boundary between non-invasive imaging and invasive angiography is shifting, but unevenly, and is best read as a series of domain-specific decisions matched to the clinical question and procedural risk. Non-invasive imaging has become the natural first investigation in several domains, while invasive angiography remains indispensable where diagnosis and treatment are inseparable. The distinctive contribution of this review is to bring the surgical perspective to a debate framed largely in imaging and interventional terms, and to argue that the next standard of care will be shaped as much by who designs the evidence as by which modality performs best, so cardiac surgeons should help shape the questions, endpoints, and trials. Ultimately, heart-team decisions should rest on the clinical domain, patient complexity, imaging quality, and whether diagnosis and treatment are separable.

## Figures and Tables

**Table 1:** Major trials and registries relevant to the invasive and non-invasive boundary.

Trial/Study	Year	Population (n)	Comparison	Key Finding	Relevance to the Boundary
SCOT-HEART	2018	Stable chest pain (4146)	CCTA + standard care vs. standard care alone	41% reduction in composite of coronary heart disease death or non-fatal MI at 5 years (HR 0.59)	CCTA-led pathway linked to improved clinical outcomes
ISCHEMIA	2020	Moderate to severe ischemia (5179)	Invasive vs. conservative strategy; blinded CCTA used for anatomic eligibility in ~75% of patients	No overall benefit of routine invasive strategy on the primary composite	CCTA used as anatomic gatekeeper for randomization
DISCHARGE	2022	Stable chest pain, intermediate pretest probability (3561)	CCTA-first vs. invasive coronary angiography-first	Non-inferior MACE at 3.5 years; major procedure-related complications 0.5% vs. 1.9%	CCTA is a safe first-line modality in this population
PRECISE	2023	Stable suspected CAD (2103)	Precision strategy (PROMISE risk tool + CCTA) vs. usual testing	Composite of invasive coronary angiography without obstructive CAD or death/MI reduced 71% (HR 0.29); invasive coronary angiography without obstructive CAD 2.6% vs. 10.2%	Risk-stratified CCTA reduces low-yield invasive procedures
BYPASS-CTCA	2023	Prior CABG, referred for invasive coronary angiography (688)	CCTA before invasive coronary angiography vs. standard care	Shorter procedure time, lower contrast volume, reduction in additional invasive testing	Imaging before invasive coronary angiography improves procedural efficiency in prior-CABG patients
FASTTRACK CABG	2024	Stable multivessel CAD planned for CABG (114)	CCTA + CT-FFR-only planning (single-arm feasibility)	Imaging-only surgical plan executable in 99% of cases; 30-day outcomes consistent with contemporary CABG benchmarks	Feasibility signal for CCTA-only preoperative planning; needs randomized confirmation
CCTA vs. ICA before TAVI (Diller meta-analysis; Jensen cohort)	2024, 2025	TAVI candidates (7458 pooled; 240 cohort)	CCTA vs. ICA for CAD detection; selective-ICA pathway	Patient-level sensitivity 95%, NPV 97%, specificity 73%; ICA canceled in 80% with no MACE difference	Supports CCTA as a rule-out gatekeeper before TAVI; no strategy-level RCT
NOTION-3	2024	Severe AS for TAVI with significant CAD (455)	FFR-guided PCI vs. conservative management	Reduced composite of death, MI, or urgent revascularization at median 2 years; more minor bleeding	Randomized evidence on managing CAD in TAVI candidates
FAME-3	2022	Three-vessel CAD (1500)	FFR-guided PCI vs. CABG (non-inferiority design)	FFR-guided PCI did not meet prespecified non-inferiority margin vs. CABG for primary composite at 1 year (10.6% vs. 6.9%)	Supports continued role of CABG in three-vessel disease
MR-INFORM	2019	Stable angina (918)	CMR perfusion-guided vs. invasive FFR-guided strategy	Non-inferior MACE at 1 year, with fewer subsequent revascularizations in CMR arm	CMR perfusion is a credible non-invasive functional gatekeeper
CONSERVE	2019	Stable suspected CAD (1631)	CCTA-first vs. direct invasive coronary angiography	78% reduction in invasive coronary angiography	Pre-screening with CCTA reduces unnecessary invasive procedures
CT-FFR validation and registry experience	2014, 2020	Stable CAD (>5000 cumulative)	CT-FFR vs. invasive FFR/clinical management	NXT sensitivity 86%, specificity 79%; ADVANCE management change in ~67%	CT-FFR extends CCTA into the physiological domain
CRISP-CT	2018	Stable and acute CAD (3912)	Pericoronary FAI vs. standard CCTA prognostication	Pericoronary FAI predicts cardiac mortality independent of stenosis severity	Non-invasive wall-based prognostication complementary to angiographic findings

CABG, coronary artery bypass grafting; CAD, coronary artery disease; CCTA, coronary computed tomography angiography; CMR, cardiac magnetic resonance; FAI, fat attenuation index; FFR, fractional flow reserve; CT-FFR, CT-derived fractional flow reserve; MACE, major adverse cardiovascular events; MI, myocardial infarction; PCI, percutaneous coronary intervention.

**Table 2:** Domain-specific framework for first-line angiographic investigation.

Clinical Scenario	Pretest Probability of Obstructive CAD	Reasonable First-Line Investigation	Indications to Proceed to Invasive Coronary Angiography	Practice Tier and Evidence Base
Stable chest pain, no high-risk features	Low to moderate	CCTA, with CT-FFR or CMR perfusion if intermediate stenoses identified	Intermediate stenosis with positive CT-FFR, non-diagnostic CT, or persisting clinical concern	Established non-invasive first-line—RCTs (SCOT-HEART, PROMISE, DISCHARGE, PRECISE); guidelines (2021 ACC/AHA chest pain; 2024 ESC chronic coronary syndromes)
Pre-TAVR planning	Any (procedural planning); low-to-moderate, ≤50% (coronary rule-out)	CCTA for annulus and access route; coronary rule-out when likelihood ≤50% and image quality adequate	High CAD likelihood, calcification or artifact limiting coronary evaluation, inconclusive CCTA, or PCI planned before TAVR	Established non-invasive first-line—guideline-endorsed (2025 ESC/EACTS); coronary rule-out from diagnostic-accuracy meta-analysis and observational selective-ICA cohorts; no strategy-level RCT
Pre-SAVR or pre-mitral surgery, low-to-moderate CAD risk	Low to moderate (≤50%)	CCTA, with CT-FFR for moderate stenoses	Obstructive CAD on CCTA, heavy calcification limiting CCTA, or suspected ischemic mitral regurgitation	Established non-invasive first-line—guidelines (2025 ESC/EACTS; 2020 ACC/AHA); meta-analytic diagnostic-accuracy data
Stable multivessel CAD planned for CABG	High, stable, low anatomic complexity	Invasive coronary angiography is the established standard; CCTA + CT-FFR-only planning should currently be performed only within a research protocol	Default pathway pending randomized confirmation with surgical primary endpoints	Contested/heart-team individualized (invasive default)—single-arm feasibility only (FASTTRACK CABG)
Heavy coronary calcification limiting CCTA confidence	Any	Invasive coronary angiography preferred; PCCT may close this gap as evidence matures	–	Invasive first-line—expert opinion and observational data
Prior CABG referred for re-evaluation	High	CCTA before invasive coronary angiography, per BYPASS-CTCA	Invasive coronary angiography is generally still performed; CCTA optimizes procedural planning	Contested/heart-team individualized—RCT (BYPASS-CTCA)
LAA closure, mitral TEER, or tricuspid intervention	Variable	CT-based procedural planning	Suspected obstructive CAD that CCTA cannot exclude with adequate confidence	Established non-invasive first-line (procedural planning)—observational data and SCCT/society consensus documents
Suspected chronic total occlusion	High	CCTA for lesion length, calcification, and collaterals; invasive coronary angiography for the procedural decision	Invasive coronary angiography is the procedural step regardless of imaging-based planning	Invasive first-line—consensus/observational planning data; expert opinion
NSTE-ACS, high risk	High	Early invasive coronary angiography (within 24 h)	–	Invasive first-line—guidelines (2023 ESC ACS), Class IIa
NSTE-ACS, very high risk	Very high	Immediate invasive coronary angiography	–	Invasive first-line—guidelines (2023 ESC ACS), Class I
STEMI or cardiogenic shock	Very high	Emergent invasive coronary angiography	–	Invasive first-line—guidelines, Class I
Aortic dissection or aneurysm	Any	CT angiography (or CMR for surveillance in connective tissue disease)	Concomitant coronary assessment or procedural fluoroscopy required	Established non-invasive first-line—guidelines (2014 ESC; 2022 ACC/AHA aortic disease)

The framework reflects the authors’ synthesis of contemporary evidence and is a starting point for multidisciplinary heart-team discussion rather than a prescriptive algorithm. CABG, coronary artery bypass grafting; CCTA, coronary computed tomography angiography; CT-FFR, CT-derived fractional flow reserve; LAA, left atrial appendage; NSTE-ACS, non-ST-elevation acute coronary syndrome; PCI, percutaneous coronary intervention; SAVR, surgical aortic valve replacement; STEMI, ST-elevation myocardial infarction; TAVR, transcatheter aortic valve replacement; TEER, transcatheter edge-to-edge repair.

## Data Availability

No new data were created or analyzed in this study. Data sharing is not applicable to this article.
